# Driver behavior profiling: An investigation with different smartphone sensors and machine learning

**DOI:** 10.1371/journal.pone.0174959

**Published:** 2017-04-10

**Authors:** Jair Ferreira, Eduardo Carvalho, Bruno V. Ferreira, Cleidson de Souza, Yoshihiko Suhara, Alex Pentland, Gustavo Pessin

**Affiliations:** 1 Applied Computing Lab, Instituto Tecnológico Vale, Belém – PA, Brazil; 2 Institute of Exact and Natural Sciences, Federal University of Pará, Belém – PA, Brazil; 3 SENAI Institute of Innovation – Mineral Technologies, Belém – PA, Brazil; 4 Recruit Institute of Technology, Mountain View – CA, United States of America; 5 Media Lab, Massachusetts Institute of Technology, Cambridge – MA, United States of America; West Virginia University, UNITED STATES

## Abstract

Driver behavior impacts traffic safety, fuel/energy consumption and gas emissions. Driver behavior profiling tries to understand and positively impact driver behavior. Usually driver behavior profiling tasks involve automated collection of driving data and application of computer models to generate a classification that characterizes the driver aggressiveness profile. Different sensors and classification methods have been employed in this task, however, low-cost solutions and high performance are still research targets. This paper presents an investigation with different Android smartphone sensors, and classification algorithms in order to assess which sensor/method assembly enables classification with higher performance. The results show that specific combinations of sensors and intelligent methods allow classification performance improvement.

## 1 Introduction

Driver behavior strongly impacts traffic security [1] and causes the vast majority of motor vehicle accidents [2]. In 2010, the total economic cost of motor vehicle crashes in the United States was 242 billion [3]. This figure represents the costs for approximately 33 thousand fatalities, 4 million nonfatal injuries, and 24 million damaged vehicles. Driver behavior adaptations might increase overall security and lessen vehicle fuel/energy consumption and gas emissions [4, 5]. In this context, driver behavior profiling tries to better understand and potentially improve driver behavior, leveraging a safer and more energy aware driving.

*Driver monitoring and analysis* or *driver behavior profiling* is the process of automatically collecting driving data (e.g., speed, acceleration, breaking, steering, location) and applying a computational model to them in order to generate a safety score for the driver. Driving data collection may be achieved by several kinds of sensors, from the general ones in smartphones, to dedicated equipment such as monitoring cameras, telematics boxes, and On-Board Diagnostic (OBD) adapters.

Modern smartphones provide sensors suitable to collect data for driver profile analysis. Previous work [6–8] shows that properly preprocessed and handled smartphone sensors data are an interesting alternative to conventional *black boxes* for the monitoring of driver behavior.

Driver behavior profiling relevance has grown in the last few years. In the insurance telematics domain, plans such as Usage-Based Insurance (UBI) or Pay-How-You-Drive (PHYD) make car insurance cheaper by rewarding drivers with good driving scores, instead of only considering group based statistics (e.g., age, gender, marital status) for that end. In the freight management domain, automated, continuous, and real-time driver behavior profiling enables managers to institutionalize campaigns aiming to improve drivers score, and, as a consequence, decrease accidents, and increase resource economy, and vehicle lifetime. Furthermore, notifications of unsafe driving events presented to drivers in real-time can help prevent accidents. For example, a smartphone app may notify the driver every time she performs an aggressive turn.

Several driver behavior profiling work [9–14] use a smartphone based sensor-fusion to identify aggressive driving events (e.g., aggressive acceleration, aggressive break) as the basis to calculate driver score. Another work [15] uses vehicle sensor data to provide driving tips and assess fuel consumption as a function of driver profile. The machine learning algorithms (MLAs) employed in these papers come down to fuzzy logic or variations of Dynamic Time Warping (DTW). Dynamic Time Warping is an algorithm to find similar patterns in temporal series. It was originally employed in the speech recognition problem [16]. We believe that other MLAs and sensor combination can be applied to the task of identifying aggressive driving events with promising results. In this context, to the best of our knowledge, there is no work that quantitatively assesses and compares the performances combinations of smartphone sensor and MLAs in a real-world experiment.

The main contribution of this work is to evaluate the performance of multiple combinations of machine learning algorithms and Android smartphone sensors in the task of detecting aggressive driving events in the context of a real-world experiment. We perform a data-collecting phase, driving a car, and gathered data from several different sensors while performing different maneuverers. We present how the machine learning methods can be employed in the task and evaluate the accuracy of the combination of sensors and technique aiming to find the best match of sensor/technique to each class of behavior.

The remainder of this paper is organized as follows. In Section 2 we present a comprehensive set of works that are related to our proposal, followed by Section 3, in which we present concepts of the employed techniques. Section 4 describes the methodology, presenting the data-gathering phase and the details of how we model the proposed machine learning application. It is followed by results and discussion (Section 5). Finally, we conclude the paper presenting the conclusions and pointing out potential future work.

## 2 Related work

In this section we describe recent driver behavior profiling work. It is worth noting that several driver behavior profiling solutions are commercially available mowadays, mostly in the insurance telematics and freight management domains. Examples include Aviva Drive (www.aviva.co.uk/drive), Greenroad (greenroad.com), Ingenie (www.ingenie.com), Snapshot (www.progressive.com/auto/snapshot), and SeeingMachines (www.seeingmachines.com). However, technical details of these solutions are not publicly available.

Nericell, proposed by Mohan et al. [17], is a Windows Mobile smartphone application to monitor road and traffic conditions. It uses the smartphone accelerometer to detect potholes/bumps, and braking events. It also employs the microphone to detect honking, and the GPS/global system of mobile (GSM) communications to obtain vehicle localization and speed. Braking, bumps and potholes are detected by comparing a set of empirically predefined thresholds to abrupt variations of accelerometer data or to their mean over a sliding window of N seconds. No MLA is employed in the detection of such events. Some event detection results in terms of False Positives (FPs) and Fale Negatives (FNs) include: 4.4% FN, and 22.2% FP for breaking events; 23% FN, and 5% FP for bumps/potholes detection at low speed (<25 kmph); and 0% FN and FP for honk detection on an exposed vehicle (e.g., a motorbike).

Dai and colleagues [9] propose an Android application aimed at real time detection and alert of dangerous driving events typically related to Driving Under the Influence (DUI) of alcohol. The application uses the smartphone accelerometer and orientation (yaw, pitch, and roll angles) sensors to detect Abnormal Curvilinear Movements (ACM) and Problems in Maintaining Speed (PMS), which are the two main categories of drunk driving related behaviors. A series of equations are used to determine lateral and longitudinal acceleration vectors. An ACM event is detected if the difference between maximum and minimum values of lateral acceleration within a 5 seconds time window exceeds an empirical threshold. A PMS is detected if longitudinal acceleration exceeds positive or negative fixed empirical thresholds at any given time. Similarly to [17], no MLA is employed for event detection. Experimental results include: 0% FN, and 0.49% FP for abnormal curvilinear movements; and 0% FN, and 2.90% FP for problems of speed control.

An iPhone application called MIROAD was created by Johnson and Trivedi [10]. MIROAD uses a smartphone based sensor-fusion of magnetometer, accelerometer, gyroscope, and GPS to detect aggressive driving events and accordingly classify driver’s style into aggressive or nonaggressive. Aggressive events are detected by a single classifier based on the DTW algorithm. All processing is executed in real-time on the smartphone. Experimental analysis shows that 97% of the aggressive events were correctly detected.

WreckWatch, proposed by White et al. [18], is an Android smartphone-based client/server application to detect car accidents. The client application detects accidents, records related data, and sends them to the server application which can notify relevant authorities. WreckWatch client uses data from the accelerometer, GPS, and microphone, with threshold-based filtering to detect an accident. The accident prediction framework is composed of a 11-tuple model of the phone state, and a function that evaluates the model to signal if it represents an accident. Model variables include the maximum acceleration experienced in any direction, and an indication if a loud sound has occurred. One scenario that triggers accident detection is when acceleration, sound, and vehicle speed are all higher than empirical thresholds. Similarly to [9, 17], no MLA is employed for accident detection. Experiment results show that dropping a phone is unlikely to cause FPs, some accidents may not be detected with smartphones, and acoustic data is not enough to detect accidents.

Araujo and colleagues [15] present a smartphone application to evaluate fuel consumption efficiency as a function of driver behavior. The application also provides real-time driving hints such as *shift gear earlier* and *accelerating too high*. Instead of collecting data from smartphone sensors, this application uses an OBD Bluetooth adapter and the Torque Pro smartphone app to collect data (e.g., speed, acceleration, RPM) from vehicle sensors. After data collection, the app extracts a series of features and applies three classifiers (one linear discriminant and two fuzzy systems) to them. All processing is executed in real-time on the smartphone.

Eren et al. [11] propose an iPhone application to classify driver behavior as either safe or risky based on risky driving events. The application detects sudden turns, lane departures, braking and acceleration events. The sensors used for event detection are the smartphone accelerometer, gyroscope, and magnetometer. The application uses the endpoint detection algorithm to demarcate start and end times of an event. The demarcated event is then compared with template event data by means of the DTW algorithm. Finally, a Bayesian classifier labels a driver’s behavior as safe or risky based on the number of events over time. Experimental results show that 14 out of 15 drivers (93.3%) were correctly classified. It is worth noting that the paper only provides driver classification results. Hence, event classification performance results are not provided.

The work by Fazeen and colleagues [19] uses an Android smartphone accelerometer and GPS to identify vehicle conditions (speed and shifting), driving patterns (acceleration/deceleration and changing lanes), and road condition (smooth, uneven, rough, or containing a bump or pothole). Events are mainly detected by calculating the time duration, difference, and slope between successive accelerometer readings on certain axes and comparing them to empirical fixed/dynamic thresholds. For example, the work states that safe acceleration and deceleration never reach a g-force of more than ±0.3*g* on *y*-axis. Similarly to [9, 17, 18], no MLA is employed for event detection. Experimental results include the following road anomaly classification accuracy: 81.5% for bumps, 72.2% for potholes, 75% for rough roads, 91.5% for smooth roads, and 89.4% for uneven roads.

Castignani et al. [12] propose a driver behavior profiling mobile tool based on fuzzy logic that makes use of accelerometer, magnetometer, and gravity sensors in Android smartphones. This tool classifies driver behavior as *Normal*, *Moderate*, and *Aggressive*, which correspond to a driving score between 0 (best) and 100 (worst). The classification and score are not processed in real-time as sensor data are collected by *UBI-Meter* mobile application and stored locally on the smartphone. Later on, these data are sent to a remote server on the Internet for processing. The work by Saiprasert and collaborators [13] employs GPS, accelerometer, and magnetometer smartphone sensors to profile driver behavior as *Very Safe*, *Safe*, *Aggressive*, and *Very Aggressive*. This profile is calculated in real-time by detecting relevant driving events. Event detection is performed by a variation of the DTW algorithm [20].

SenseFleet, proposed by Castignani et al. [14], is a driver behavior profiling platform for Android smartphones that is able to detect risky driving events independently from the mobile device and vehicle. The mobile application collects data from accelerometer, magnetometer, gravity sensor, and GPS smartphone sensors and makes use of a fuzzy system to detect risky events such as excessive speed, turning, acceleration, and breaking that might occur in a trip. The application calculates a driving score between 0 (worst) and 100 (best) for each trip as a function of detected risky events. All processing is done in real-time on the smartphone. Authors performed several experiments. In one of these experiments, more than 90% of risky events were correctly identified by the application.

Wahlstrom, Skog and Handel [21] propose a framework for the detection of dangerous vehicle cornering events based on the theoretical likelihood of tire slippage (slipping) and vehicle rollover. The Global Navigation Satellite System (GNSS)—a general term for GPS—of Android smartphones is the sole sensor used for event detection. By employing classical mechanics equations, theoretical thresholds for slipping and vehicle rollover are defined. The slipping threshold is defined with respect to tangential and rotational velocities, tangential acceleration, and the coefficient of friction between the vehicle’s tires and the road. The vehicle rollover threshold is defined with respect to the vehicle’s center of gravity, track width, and height. Similarly to [9, 17–19], no MLA is employed in event detection. Experimental analysis with GNSS data collected from three different Android smartphone showed an average rate of 13% for both FP and FN as best results.

We have identified some noteworthy points in related work presented here: (i) all papers—except [21]—use sensor-fusion data as input for the event detection algorithms; (ii) the employed MLAs come down to fuzzy logic [12, 14, 15] or variations of DTW [10, 11, 13]; (iii) most recent papers make use of smartphones sensors instead of vehicle or telematics boxes sensors; and (iv) some papers [9, 17–19, 21] do not use MLAs to detect driving events, instead they employ physical equations or fixed/dynamic thresholds or a combination of both to this end. In our work, we run the machine learning algorithms locally, although, future versions might run on a cloud-based fashion, as presented in [22, 23]. Other approaches related to driver behavior are related to social mechanisms, like the evaluation performed by Song and Smith [24], in which driver behavior is investigated in a high-occupancy toll lane. A more comprehensive approach about smart and connected communities can be seen in the work by Sun and colleagues [25]. For a more thorough comparison of smartphone-based sensing in vehicles please refer to the work by Engelbrecht and colleagues [26].

## 3 Machine learning algorithms

In our evaluation, we compare the performance of four MLAs: Artificial Neural Networks (ANN), Support Vector Machines (SVM), Random Forest (RF), and Bayesian Network (BN). Those MLAs were chosen given their great presence in the literature of classification problems, and the fact that they represent different machine learning “tribes” [27], which ensures a machine learning algorithmic diversity. In this section, basic concepts of the aforementioned MLAs are explained.

### 3.1 Artificial neural networks

Artificial Neural Networks (ANN) are composed by several computational elements that interact through connections with different weights. With inspiration in the human brain, neural networks exhibit features such as the ability to learn complex patterns of data and generalize learned information [28]. The simplest form of an ANN is the Multi Layer Perceptron (MLP) consisting of three layers: the input layer, the hidden layer, and the output layer.

Haykin [29] states that the learning processes of an artificial neural network are determined by how parameter changes occur. Thus, the process of learning an ANN is divided into three parts: (i) the stimulation by extraction of examples from an environment; (ii) the modification of its weights through iterative processes in order to minimize ANN output error; and (iii) the network responds in a new way as a result of the changes that occurred. Parameter configuration directly impacts on the process of learning an ANN. Some examples of parameters are: learning rate, momentum rate, stop criteria and form of network training.

### 3.2 Support vector machines

Support Vector Machines (SVM) [30] are a supervised learning method used for regression and classification. The algorithm tries to find an optimal hyperplane which separates the *d*-dimensional training data perfectly into its classes. An optimal hyperplane is the one that maximizes the distance between examples on the margin (border) which separates different classes. These examples on the margin are the so-called “support vectors”.

Since training data is often not linearly separable, SVM maps data into a high-dimensional feature space though some nonlinear mapping. In this space, an optimal separating hyperplane is constructed. In order to reduce computational cost, the mapping will be performed by kernel functions, which depend only on input space variables. The most used kernel functions are: linear, polynomial, radial base function (RBF) and sigmoid.

### 3.3 Random forrest

Random Forests (RF) are sets of decision trees that vote together in a classification. Each tree is constructed by chance and selects a subset of features randomly from a subset of data points. The tree is then trained on these data points (only on the selected characteristics), and the remaining “out of bag” is used to evaluate the tree. Random Forests are known to be effective in preventing overfitting.

Proposed by Leo Breiman [31] its features are: (i) it is easy to implement; (ii) it has good generalization properties; (iii) its algorithm outputs more information than just class label; (iv) it runs efficiently on large data bases; (v) it can handle thousands of input variables without variable deletion; and (vi) it provides estimates of what variables are important in the classification.

### 3.4 Bayesian networks

According to Ben-Gal [32], Bayesian Networks (BNs) belong to the family of probabilistic graphical models. These graph structures are used to represent knowledge about an uncertain domain. In particular, each node in the graph represents a random variable, while the edges between the nodes represent probabilistic dependencies among the corresponding random variables. Such conditional dependencies in the graph are often estimated using known statistic and computational methods. Thus, Bayesian networks combine principles of graph theory, probability theory, and statistics.

## 4 Methodology

We modeled this work as a multi-label supervised learning classification problem where the labels are driving events types. The goal of this work is to identify the best combination of motion sensor (and its axes), learning algorithm (and its parameters), and number of frames in the sliding window (*nf*) to detect individual driving event types. To this end, we define an evaluation assembly in the form *EA = {1:sensor, 2:sensor axis(es), 3:MLA, 4:MLA configuration, 5:nf*}.

An assembly is evaluated by training, testing, and assessing the performance of the classifier generated by the specified MLA (element #3) with its configuration parameters (element #4) over a data set identified by sensor (element #1), its axis(es) (element #2), and number of frames in sliding window (element #5). By changing the value of an element in this assembly, we achieve a different driving event detection performance. Therefore, this assessment evaluates several combinations of element values in order to reveal the best performing ones for each driving event type.

**Fig 1:**
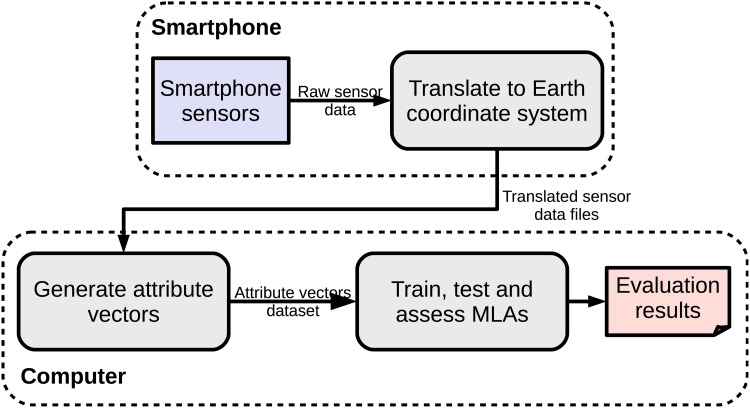
High level view of our evaluation pipeline showing processing steps from raw sensor data sampling to training, testing, and assessing MLAs.

**Fig 2:**
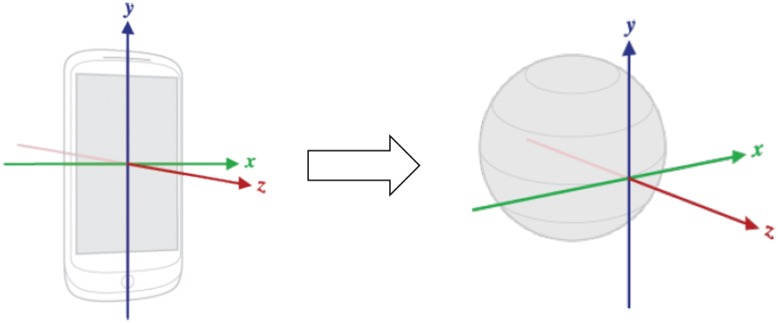
Sensor data are translated from the device coordinate system to Earth’s, in order to achieve device position independence.

Fig 1 shows a high level view of our evaluation pipeline. In the first step of the pipeline, smartphone sensor raw data is sampled and translated from the device coordinate system to Earth’s coordinate system (Fig 2). This translation is necessary in order to achieve device position independence inside the vehicle. Translated sensor data are then stored in the smartphone file system. In the second step, translated sensor data files are retrieved from the smartphone and used as input to generate attribute vector data sets. In the third step, attribute vector data sets are used to train, test and assess MLAs performances. As depicted in Fig 1, the first pipeline step is executed on the smartphone, whereas the second and final steps are executed on a regular computer.

The metric we use to evaluate assembly performance for each driving event type is the area under the ROC curve (AUC) [33, 34]. The AUC of a classifier ranges from 0.0 (worst) to 1.0 (best), but no realistic classifier should have an AUC less than 0.5 which is equivalent to random guessing. Hence, the closer an evaluation assembly AUC is to 1.0, the better it is at detecting a particular driving event type.

In the remainder of this section, Subsection 4.1 presents the detailed evaluation assembly of machine learning and sensors. Subsection 4.2 describes the proposed attribute vector, used as input for the machine learning algorithms. Finally, Subsection 4.3 presents how we performd the data collection in a real-world experiment.

### 4.1 Evaluation assembly

The *sensor* is the first element of the evaluation assembly. It represents one of the following Android smartphone motion sensors: accelerometer (Acc), linear acceleration (LinAcc), magnetometer (Mag), and gyroscope (Gyr). The accelerometer measures the acceleration in meters per second squared (*m*/*s*^2^) applied to the device, including the force of gravity. The linear acceleration sensor is similar to the accelerometer, but excluding the force of gravity. The magnetometer measures the force of the magnetic field applied to the device in micro-Tesla (*μ*T), and works similar to a magnet. The gyroscope measures the rate of rotation around the device’s axes in radians per second (*rad*/*s*). These sensors provide a 3-dimensional (*x*, *y* e *z*) temporal series with nanoseconds precision in the standard sensor coordinate system (relative to the device).

The second element of the evaluation assembly is the sensor axis(es). Available values for this element are (i) all 3 axes; (ii) *x* axis alone; (iii) *y* axis alone; and (iv) *z* axis alone. For example, the accelerometer originates the following data sets: *accelerometer* (with data from all three axes), *accelerometer_x*, *accelerometer_y*, and *accelerometer_z*. The only exception to this rule is the magnetometer whose *x* axis values are always 0 or close after translated to Earth’s coordinate system. For that reason, there is no *magnetometer_x* data set. As we evaluate data from 3 sensors that originate 4 data sets, and 1 sensor that originates 3 data sets, there is a total of 3 * 4 + 3 = 15 data sets. We separated sensor axes in distinct data sets to observe if any single axis would emerge as the best to detect a particular driving event type.

The MLA is the third element of the evaluation assembly. As detailed in Section 3, we evaluate the classification performance of MLP, SVM, RF, and BN MLAs. We used the WEKA (version 3.8.0) implementations of these algorithms in conjunction with LIBSVM [35] library (version 3.17). We trained and tested these classifiers using 10-fold cross-validation in order to minimize overfitting.

Algorithm configuration is the forth element of the evaluation assembly. We performed a parameter grid search to assess each algorithm with every possible combination of parameter values on Table 1. We set most of the parameter values experimentally, and followed the guidelines provided in [36] for SVM. We also used WEKA default values for parameters not listed on Table 1.

**Table 1:** MLAs configurations.

Algorithm	Parameter	Values
BN	Search algorithm	K2, Repeated Hill Climber
	Conditional probability estimator algorithm	Simple (directly from data), Bayes Model Averaging [37]
MLP	# of single hidden layer neurons	(#*attr*. + #*classes*)/2, 40, 30, 20, 10
RF	# of iterations	200, 100
	# of attributes to randomly investigate	*log*_2_(#*predictors*) + 1, 10, 15
SVM	Kernel function	linear, polynomial, radial basis function, sigmoid
	*C*	2^−3^, 2^−1^, 2
	γ	2^−13^, 2^−11^, 2^−9^

**Fig 3:**
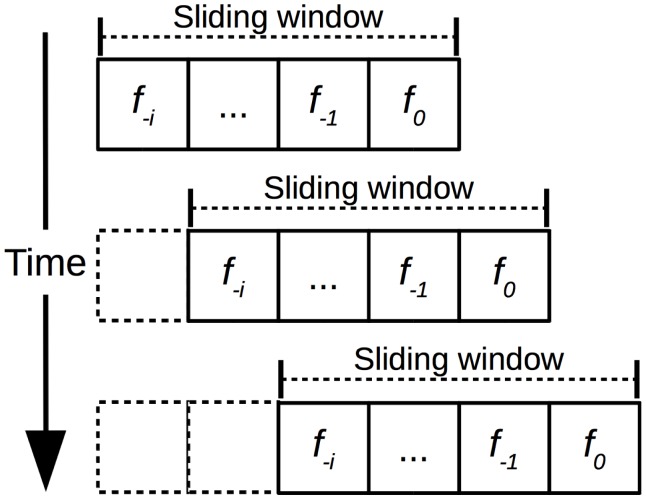
Time window composed of *nf* one-second frames which group raw sensor data samples. The time window slides in 1 frame increments as time passes. *f*_0_ is the frame of the current second, *f*_−1_ is the frame of the previous second, and so forth down to *f*_−*i*_, where *i* = *nf* − 1.

Raw sensor data are basically composed of 3-axes values and a nanosecond timestamp indicating the instant the sample was collected. However, we do not send raw sensor data to classifiers. Instead, we group sensor time series samples in one-second length frames to compose a sliding time window which is later summarized to originate an attribute vector. As time passes, the window is slided in 1 frame increments over the temporal series as depicted in Fig 3. We consider *f*_0_ as the frame of the current second, *f*_−1_ as the frame of the previous second, and so forth down to *f*_−(*nf* − 1)_, where *nf* (the fifth element of the evaluation assembly) is the number of frames that compose the sliding time window. We used the following *nf* values in this evaluation: 4, 5, 6, 7, and 8. These values were defined experimentally so that the sliding window can accommodate the length of collected driving events which range from 2 to 7 seconds depending on how aggressive the event is.

In this work, we assessed the performance of several evaluation assemblies to find the ones that best detect each driving event type. The number of assemblies is the result of all combinations of 15 data sets, 5 different values for *nf*, 4 configurations (Table 1) of the BN algorithm, 5 of the MLP, 6 of the RF, and 36 of the SVM. This results in a total of 15 * 5 * 4 + 15 * 5 * 5 + 15 * 5 * 6 + 15 * 5 * 36 = 3825 evaluation assemblies.

### 4.2 Attribute vector

An attribute vector is the summarization of the sliding window depicted in Fig 3. One instance of the attribute vector is generated for every time window that contains a driving event on it. Correspondingly, if there is no driving event for a particular time window, no attribute vector instance is generated.

**Fig 4:**
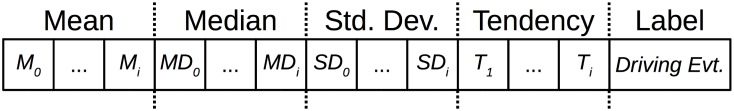
Attribute vector summarizing a sliding time window of*nf* frames, *i* = *nf* − 1.

We create an instance of the attribute vector by calculating the mean (*M*) Eq (1), median (*MD*) Eq (2), standard deviation (*SD*) Eq (3), and the increase/decrease tendency (*T*) Eq (4) over sensor data samples in the frames composing the time window. The number of attributes in the vector is dependent on the number of frames in the sliding window (*nf*). There are *nf* mean, median, and standard deviation attributes, and *nf* − 1 tendency attributes. Fig 4 depicts the structure of an attribute vector for a single axis of sensor data. When the data set is composed of more than one axis, the attribute vectors for each axis are simply concatenated and only the class label attribute of the last vector is preserved as they all have the same value.

The attributes of the vector are calculated as:

$$M_{0}=M\left(f_{0}\right)\;\cdots \;M_{i}=M\left(f_{-i},f_{0}\right)$$ (1)

$$MD_{0}=MD\left(f_{0}\right)\;\cdots \;MD_{i}=MD\left(f_{-i},f_{0}\right)$$ (2)

$$SD_{0}=SD\left(f_{0}\right)\;\cdots \;SD_{i}=SD\left(f_{-i},f_{0}\right)$$ (3)

$$T_{1}=\frac{M(f_{-1})}{M(f_{0})}\;\cdots \;T_{i}=\frac{M(f_{-i})}{M(f_{0})}$$ (4)

Where *i* = [0..(*nf* − 1)], *SF*(*f*_*j*_) is a summarizing function (mean, median, or standard deviation) applied over the samples of the *j*^*th*^ frame, and *SF*(*f*_*j*_, *f*_*k*_) is a summarizing function applied over the samples from the *j*^*th*^ to the *k*^*th*^ frame (*j* < *k*).

The class label attribute of the vector comes from the driving events ground-truth, as described later in Section 4.3. The class label is the driving event whose start timestamp is between the first timestamp of the samples in frame *f*_−(*nf* − 1)_ and the last timestamp of the samples in frame *f*_0_. We should mention that attribute vectors originating from different driving trips can be grouped together in the same data set as they are time-independent.

It is important to highlight that a single driving event raw sensor data sample generates *nf* attribute vector instances. This occurs because the window frame that contains the start timestamp of the event changes its position in the window as it slides. Therefore, if there are *s* samples of a particular driving event type, there will be *s* * *nf* attribute vector instances for that same event type. This behavior allows for multiple windows to capture different portions or signatures of the same event.

### 4.3 Data collection in a real-world experiment

We performed a real-world experiment in order to collect sensor data for driving events. In this experiment, an Android application recorded smartphone sensor data while a driver executed particular driving events. We also recorded the start and end timestamps of the driving events to generate the ground-truth for the experiment.

We performed the experiment in 4 car trips of approximately 13 minutes each in average. The experiment conditions were the following: (i) the vehicle was a 2011 Honda Civic; (ii) the smartphone was a Motorola XT1058 with Android version 5.1; (iii) the smartphone was fixed on the car’s windshield by means of a car mount, and was neither moved nor operated while collecting sensor data; (iv) the motion sensors sampling rate varied between 50 and 100 Hz, depending on the sensor; (v) two drivers with more than 15 years of driving experience executed the driving events; and (vi) the weather was sunny and the roads were dry and paved with asphalt.

**Fig 5:**
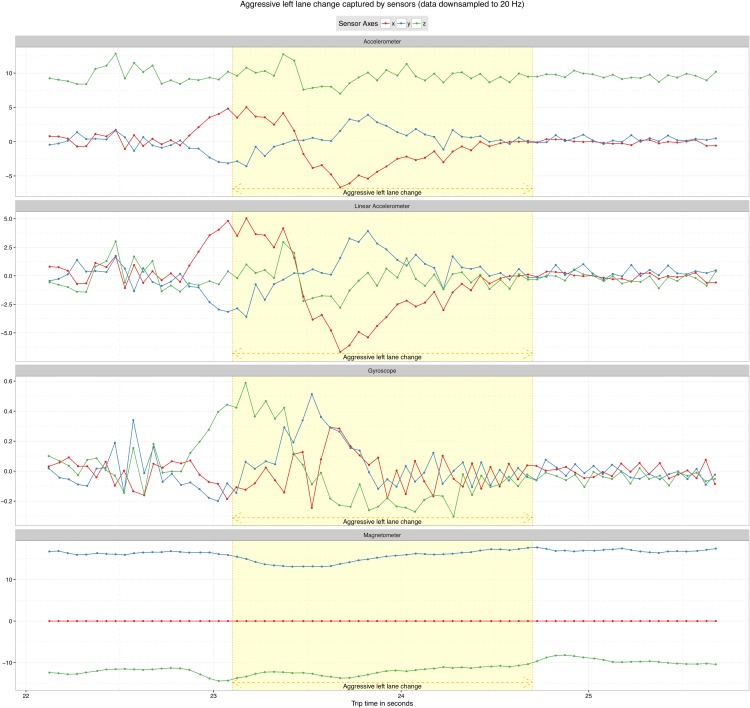
Aggressive lane change event data captured by the four sensors used in this evaluation.

The driving events types we collected in this experiment were based on the events in [13]. Our purpose was to establish a set of driving events that represents usual real-world events such as breaking, acceleration, turning, and lane changes. Table 2 shows the 7 driving events types we used in this work and their number of collected samples. Fig 5 shows sensor data for an aggressive left lane change event as it is captured by the four sensors used in this evaluation.

**Table 2:** Driving event types and number of samples.

Driving Event Type	# of samples
Aggressive breaking	12
Aggressive acceleration	12
Aggressive left turn	11
Aggressive right turn	11
Aggressive left lane change	4
Aggressive right lane change	5
Non-aggressive event	14
**Total**	**69**

## 5 Results

**Fig 6:**
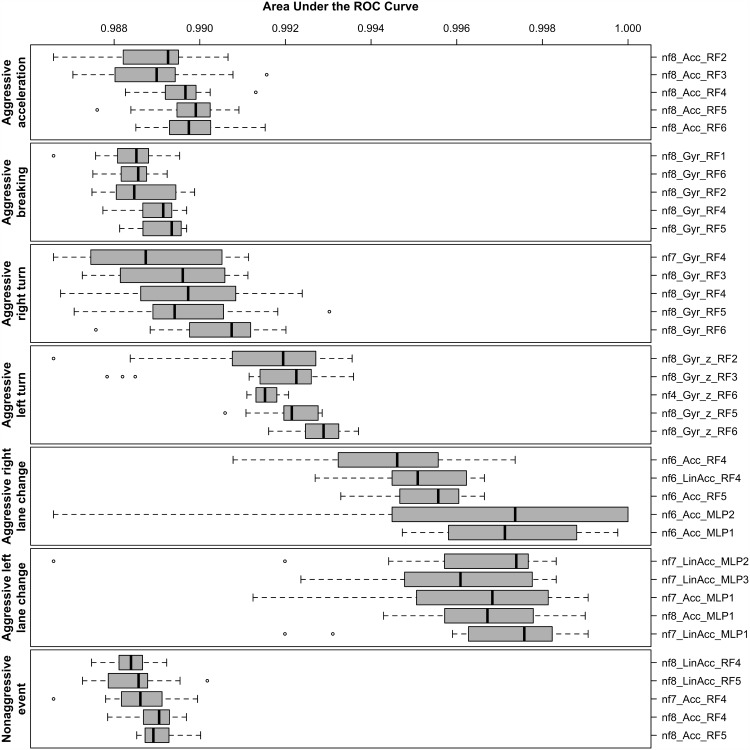
Top 5 best AUC assemblies grouped by driving event type as the result of 15 MLA train/test executions with different random seeds. Values closer to 1.0 are better. Driving events are on the left-hand side and assemblies are on the right-hand side. Assemblies with the best mean AUC are closer to the bottom.

We executed all combinations of the 4 MLAs and their configurations described on Table 1 over the 15 data sets described in Section 4.3 using 5 different *nf* values. We trained, tested, and assessed every evaluation assembly with 15 different random seeds. Finally, we calculated the mean AUC for these executions, grouped them by driving event type, and ranked the 5 best performing assemblies in the boxplot displayed in Fig 6. This figure shows the driving events on the left-hand side and the 5 best evaluation assemblies for each event on the right-hand side, with the best ones at the bottom. The assembly text identification in Fig 6 encodes, in this order: (i) the *nf* value; (ii) the sensor and its axis (if there is no axis indication, then all sensor axes are used); and (iii) the MLA and its configuration identifier.

In light of the these results, we can draw a few conclusions in the context of the performed experiment. Firstly, MLAs perform better with higher *nf* values (i.e., bigger sliding window sizes). Of the 35 best performing assemblies, 23 have *nf* = 8, 6 have *nf* = 7, 5 have *nf* = 6, and only 1 has *nf* = 4.

Secondly, the gyroscope and the accelerometer are the most suitable sensors to detect the driving events in this experiment. On the other hand, the magnetometer alone is not suitable for event detection as none of the 35 best assemblies use that sensor. Also, using all sensor axes performs better in a general way than using a single axis. The only exception being the *z* axis of the gyroscope that alone best detects aggressive left turns.

Thirdly, RF is by far the best performing MLA with 28 out of 35 best assemblies. The second best is MLP with 7 best results. RF dominates the top 5 performances for nonaggressive events, and aggressive turns, breaking, and acceleration. However, MLP is better at aggressive lane changes. BN and SVM were not ranked in the best 35 performing assemblies.

Fourthly, MLP configuration #1 was the best performing. In this configuration, the number of neurons in the hidden layer is defined as (#*attr*. + #*classes*)/2. This is also the default WEKA configuration. For RF, configurations #6 (# of iterations = 200; # of attributes to randomly investigate = 15), and #5 (# of iterations = 200; # of attributes to randomly investigate = 10) gave the best results.

Finally, we found a satisfactory and equivalent performance in the top 35 ranked evaluation assemblies. This is true because the difference between the worst AUC mean (0.980 for the aggressive breaking event) and the best one (0.999 for the aggressive right lane change event) is only 0.018. A difference that is not significant in the context of this experiment.

## 6 Conclusions and future work

In this work we presented a quantitative evaluation of the performances of 4 MLAs (BN, MLP, RF, and SVM) with different configurations applied in the detection of 7 driving event types using data collected from 4 Android smartphone sensors (accelerometer, linear acceleration, magnetometer, and gyroscope). We collected 69 samples of these event types in a real-world experiment with 2 drivers. The start and end times of these events were recorded serve as the experiment ground-truth. We also compared the performances when applying different sliding time window sizes.

We performed 15 executions with different random seeds of 3865 evaluation assemblies of the form *EA = {1:sensor, 2:sensor axis(es), 3:MLA, 4:MLA configuration, 5:number of frames in sliding window*}. As a result, we found the top 5 performing assemblies for each driving event type. In the context of our experiment, these results show that (i) bigger window sizes perform better; (ii) the gyroscope and the accelerometer are the best sensors to detect our driving events; (iii) as general rule, using all sensor axes perform better than using a single one, except for aggressive left turns events; (iv) RF is by far the best performing MLA, followed by MLP; and (v) the performance of the top 35 combinations is both satisfactory and equivalent, varying from 0.980 to 0.999 mean AUC values.

As future work, we expect to collect a greater number of driving events samples using different vehicles, Android smartphone models, road conditions, weather, and temperature. We also expect to add more MLAs to our evaluation, including those based on fuzzy logic and DTW. Finally, we intend use the best evaluation assemblies observed in this work to develop an Android smartphone application which can detect driving events in real-time and calculate the driver behavior profile.
